# Inter-University Centre in Dubrovnik: 50 years of fostering science without borders

**DOI:** 10.3325/cmj.2022.63.313

**Published:** 2022-08

**Authors:** H. Joachim Seitz

**Affiliations:** 1 Member of the Inter-University Centre Executive Committee; 2 University Medical Center Hamburg-Eppendorf (UKE), Hamburg, Germany

**Figure 1:**
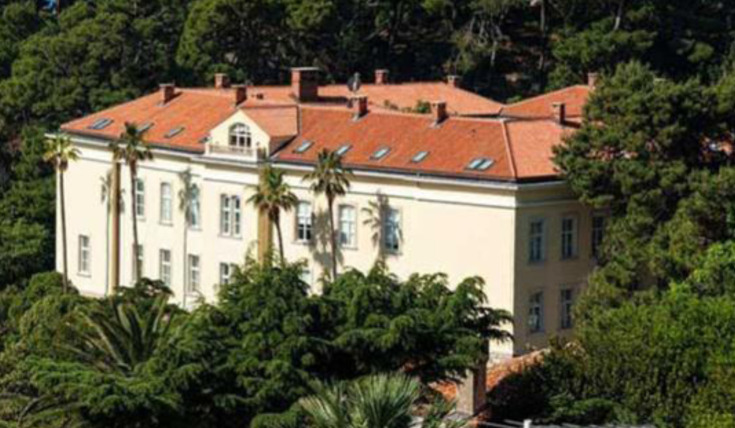
View of the Inter-University Centre, Dubrovnik, Croatia.

Its location at the Mediterranean Sea, the welcome character of its landscape and people, and over 2000 years of its rich history make Croatia one of the most privileged countries in Europe. Croatia is also the home of a special academic jewel: the Inter-University Centre (IUC) in Dubrovnik. The IUC is an independent university center established by a network of international universities with an aim to promote academic cooperation. It was founded in 1972 by 30 universities under the leadership of Ivan Supek, an outstanding physicist, survivor of a Gestapo prison, and later dean of the University of Zagreb. Two founding directors – Orjar Oyen, professor in sociology at University Bergen and Peter Fischer-Appelt, president of the University of Hamburg – have remained cordially associated to the IUC. The building where the center is located, former Don Frane Bulić high school, was donated by the City of Dubrovnik (Figure 1).

At that time of its founding, a concerned academic community saw the importance of creating new opportunities for contact, and exchange of knowledge and ideas across division lines and for addressing contemporary societal topics. There was a wish to strengthen the role of scientists in bridge-building between nations and cultures, ideologies and political systems. The former Yugoslavia, as a non-aligned country, provided conditions for contacts that could not otherwise be established. The IUC served as a bridge between East and West, and soon became a place for vibrant academic exchange. Eventually, before the outbreak of the Homeland war in 1991, when Yugoslavia broke apart and Croatia sought its independence, it encompassed more than 250 member institutions. Following the war, the number of member universities slightly decreased, but the IUC continued its mission. A part of the mission was re-connecting the academic community from the region (details see IUC Brochure 2022, www.iuc.hr). Today, the center is as vibrant and bustling as ever. In 2021, despite the coronavirus disease 2019 pandemic, the center welcomed 1543 participants and over 1000 lecturers from more than 70 countries in 38 events!

At the beginning, humanities and social sciences were in focus, but soon medicine began to contribute to the academic life and quality of the IUC. Since then many events from the field of medicine took place, just to mention a few: the Summer Stroke School (32 times!); the annual Multiple Sclerosis Academy; Public Health Approach to Mental Health; the annual conference of the International Research Group for Psycho-Societal Analysis; and the annual meetings *In the Shadow of PTSD*. Together with European Medical Societies (Immunology, Endocrinology, and Nutrition), the IUC also organized and financed summer schools for the whole Southeast Europe.

Joining IUC courses as a lecturer or a participant has been a pleasure and excitement. Just to mention a few special events organized by Prof. I. Đikić (University of Frankfurt) together with the Nobel Prize Winners in medicine: E. Fischer and J. Baltimore (both USA), and H. Michel (Germany), presenting molecular medicine advancements to medical students and young medical doctors from Zagreb, Sarajevo, Tirana, etc.

Thanks to Deutscher Akademischer Austauschdienst, after Kosovo war the IUC, together with the Zagreb University School of Medicine (Prof. D. Ježek), became a Regional Center of the Curriculum Reform in Medicine. This opened the road to the EU and consequently to the EU-financed TEMPUS Projects in Skopje, Pristina, and Belgrade (each cca. € 500 000).

Now, as Europe and the Mediterranean region are again faced with horrible conflicts, poverty, even starvation, continuing medical education based on the platform of medical sciences is needed more than ever. The IUC will continue to be a special international meeting place at the outstanding location of Dubrovnik. From September 29 – October 1, 2022, we are celebrating our anniversary under the high auspices of the President of Croatia, Mr. Zoran Milanović. The event named Fostering Inclusive Internationalization – Role and Responsibilities of Science in Addressing Global Challenges will gather outstanding international speakers (Box 1).

IUC, stay vital for another 50 years!

Box 1 **Outline of the program of the Inter-University Centre’s 50th Anniversary**

• Prof. Dr. Thomas C. Mettenleiter (Germany): Bio-medical Perspectives on the Pandemic

• Prof. Dr. Gunn Birkelund (Norway): The Social Impact of Pandemics as a Global Challenge

• Dr. Arabinda Mitra (India): Reorienting Science, Technology and Innovation (STI) Priorities to Meet the Impending Global Challenges Through Partnership – Indian Model

• Prof. Dr. Ksenija Turković (Croatia): Facing Global Challenges by Promoting Human Dignity and Respect for the Rule of Law through Education and Science

• Prof. Dr. Henk Kummeling (The Netherlands): Universities and the Future of Inclusive International Scientific Cooperation

